# The Little Book of Viruses

**DOI:** 10.1371/journal.pbio.1001139

**Published:** 2011-09-06

**Authors:** Michael Emerman

**Affiliations:** Divisions of Human Biology and Basic Sciences, Fred Hutchinson Cancer Research Center, Seattle, Washington, United States of America

## Abstract

Michael Emerman reviews science writer Carl Zimmer's latest book.

**Figure pbio-1001139-g001:**
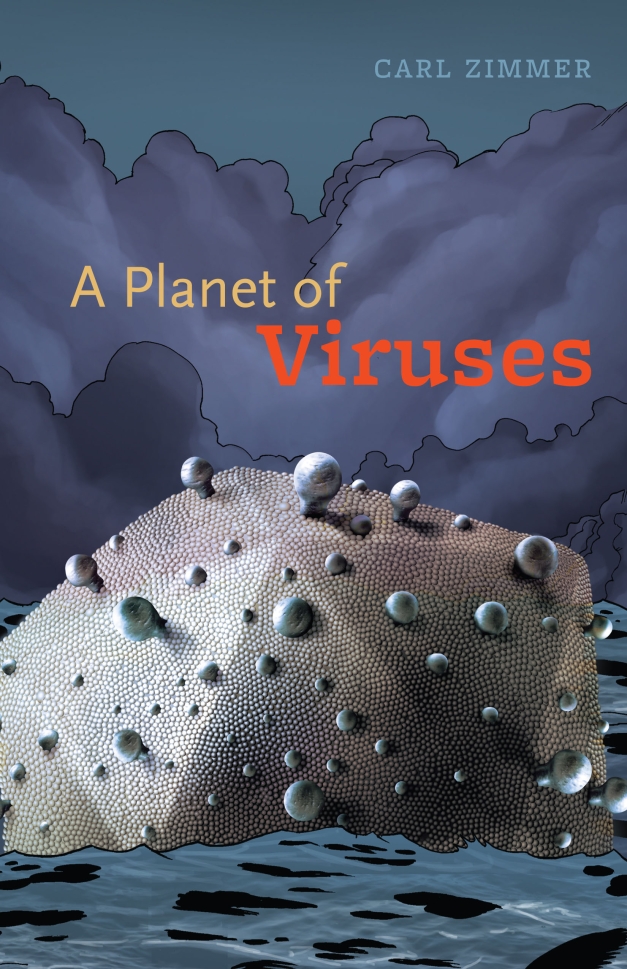
Zimmer C (2011) A Planet of Viruses. Chicago: University Of Chicago Press. 128 p. ISBN 978-0226983356 (hardcover). US**$**20.00.

The understanding of how viruses affect all life on earth has exploded in recent years. For example, it has become clear that viruses in the ocean outnumber all other organisms and that their influence on marine communities may have large-scale effects on the composition of the atmosphere. Viruses are also ancient agents of selection for evolutionary adaptation. A lively debate on viral origins argues that they predate the Last Universal Cellular Ancestor (LUCA) and may be responsible for driving the change from an RNA to a DNA world [1]. Viral disease played a critical role in shaping key events in human history—for example, smallpox epidemics killed over 300 million people in the last 700 years, including a large number of heads of state, and allowed the European conquest of the Americas. Emerging and re-emerging viral pathogens, from SARS to avian influenza to foot-and-mouth disease, continue to adversely affect our health and the viability of our food supply. Finally, a large percentage of our own DNA is actually made up of fossils of ancient viral infections that got “stuck” in our genome; so, in a sense, we are descendants of viruses as much as we are of our human ancestors.

If you already knew most of these things, then Carl Zimmer's new book, *A Planet of Viruses*, is not for you. If you didn't, then you are likely to be fascinated by this introduction to the world of viruses presented in a series of 12 short vignettes describing old viruses, new viruses, and viruses that are everywhere. The usual suspects are here—influenza, HIV, Ebola, and smallpox—but also less well-known stories such as the exciting discovery of the diversity and impact of marine viruses, the renewed interest in bacteriophages as therapy, and the fascinating story of how a giant virus found in a water tower in England was originally misinterpreted as a bacterium. This last virus, the Mimivirus, described in the book's epilogue, is in fact a virus of amoebae that has changed some of the definitions of viruses because of its large size, genomic complexity, ability to encode parts of the protein translation machinery, and its own viral parasite—all properties previously thought solely within the realm of “living” organisms [2].

Each chapter of *A Planet of Viruses* begins with some interesting historical background and ends with a thought-provoking (though often somewhat disconnected) final paragraph. Each chapter is about 5 pages long, and the whole thing is less than 80 pages of text. It's a very easy 2-hour read for anyone with a minimum amount of biology (say, high school level).

It is, however, a maddening book to read for someone who teaches virology because of the abundance of big and small mistakes throughout. For example, measles virus and smallpox, not influenza and smallpox (page 67), were likely responsible for the Native American deaths after contact with Europeans [3]. Cervical cancer is bad, but it is not the third-leading cause of death among women (page 25). It's not even the third-leading cause of cancer deaths among women (colorectal cancer claims that mantle) [4]. RNA is not the single-stranded version of DNA (page 91); they have different structures and properties. The explanation of how HIV causes AIDS is both confused and outdated (page 57)—confused because, although he correctly describes viral latency as how HIV can become silent within cells and hide from the immune system, this idea is important for understanding why it is so difficult to eradicate HIV, but has little to do with how HIV causes AIDS; outdated because the text disregards the recent paradigms coming from work on primates naturally infected with high levels of relatives of HIV that suggests chronic infection per se is not the cause of AIDS, but rather a chronic state of immune activation [5]. The sentence “each winter, 36,000 people die of the flu in the United States…” (page 16) would be correct only if the crucial words “on average during the 1990s” were included; more importantly, since the number of deaths from 1976 to 2006 ranges from a low of ∼3,000 to a high of ∼49,000 [6], the key point is that influenza seasons are unpredictable because of changing viral strains and changing levels of pre-existing immunity in the population. And the typos. Really, doesn't University of Chicago Press have a spellchecker?

I could go on and on here—in fact, my copy is filled with red X's marking places where something is not quite right. Is this important though? Many of the errors (though certainly not all) are those of simplification, and of course, too much detail can get in the way of communicating science to the public. One can forgive some errors in the service of bringing the hidden life of viruses to a broad audience. In that regard, this book is an excellent primer to get people excited about the amazing things that viruses do and why we should pay attention to them. It will likely inspire readers to learn more about how our world is influenced by past, present, and future viruses. Indeed, when I lent this book out to various younger people, they were uniformly enthusiastic with comments such as “it's short and good” and “viruses are cool!”

Viruses are fascinating because of their evolutionary dance with their hosts, because of the myriad of solutions they have come up with for replication with a limited repertoire of genes, and because of the unpredictable ways they interact with the immune system, making vaccines so difficult to rationally develop and pathogenesis so variable. They have such an influence on their environment that any biology experiment that fails to take the underlying toll of viral infections into account is necessarily flawed. Also, there is the never-ending question of whether or not they are really “living,” to which I usually reply (at least to someone below the graduate student level) that they are more like zombies—neither dead nor alive, dependent on the living for their food sources, yet capable of tricking their hosts into making more zombies. Who wouldn't want to know more about that?[Fig pbio-1001139-g001]

**About the Author**

Dr. Michael Emerman is a Member in the Divisions of Human Biology and Basic Sciences at the Fred Hutchinson Cancer Research Center in Seattle, Washington. He completed his Ph.D. at the University of Wisconsin–Madison in Cellular and Molecular Biology with Howard Temin and was a postdoctoral fellow at the Pasteur Institute in Paris. His lab studies the interaction of the human immunodeficiency virus with its host and, more recently, the effects of ancient viral infections on modern host antiviral genes. He teaches a popular graduate virology class called “Human Pathogenic Viruses.”
